# Land Use Change Reshapes Climate‐Driven Diversity Patterns of Tropical Arbuscular Mycorrhizal Fungi

**DOI:** 10.1111/mec.70253

**Published:** 2026-01-28

**Authors:** Justin D. Stewart, Dario X. Ramirez, Antonio Leon‐Reyes, Noelia Barriga, Sol Llerena, Bethan F. Manley, Natalia Carpintero‐Salvador, Melany Ruiz‐Uriguen, Jos M. Raaijmakers, E. Toby Kiers, James T. Weedon

**Affiliations:** ^1^ Amsterdam Institute for Life and Environment (A‐LIFE), Section Ecology & Evolution Vrije Universiteit Amsterdam Amsterdam the Netherlands; ^2^ Society for the Protection of Underground Networks (SPUN) Wilmington Delaware USA; ^3^ Laboratorio de Biotecnología Agrícola y de Alimentos, Ingeniería en Agronomía, Colegio de Ciencias e Ingenierías Universidad San Francisco de Quito USFQ, Campus Cumbayá Quito Ecuador; ^4^ Core Lab de Ciencias Ambientales, Decanato de Investigación Universidad San Francisco de Quito USFQ Quito Ecuador; ^5^ Departamento de Biología, Colegio de Ciencias Biológicas y Ambientales Universidad San Francisco de Quito USFQ Quito Ecuador; ^6^ Department of Microbial Ecology Netherlands Institute of Ecology Wageningen the Netherlands; ^7^ Institute of Biology Leiden University Leiden the Netherlands; ^8^ Amsterdam Institute for Life and Environment (A‐LIFE), Section Systems Ecology Vrije Universiteit Amsterdam Amsterdam the Netherlands

**Keywords:** arbuscular mycorrhizal fungi, biodiversity, climate, land use, tropical mountain ecosystem

## Abstract

Land use change and agricultural expansion threaten biodiversity yet the effects on soil life remain poorly understood, especially for microbes. Arbuscular mycorrhizal (AM) fungi are microbes that form associations with most plant species and are essential for plant nutrient uptake. The diversity of these fungi is also sensitive to both land use change and regional climatic conditions. We therefore asked whether variation in AM fungal diversity is driven by land use change, and whether these effects are further influenced by interactions with temperature and precipitation gradients. To test this, we quantified AM fungal biodiversity in cultivated and adjacent uncultivated soils across a 1700 m elevational gradient (temperature: 7.7°C–16.5°C and precipitation: 1000–3500 mm). We found that conversion of uncultivated soils to agriculture reduced AM fungal richness by 80%, on average. Richness in uncultivated soils increased with the temperature gradient, while richness in farms declined. A similar but inverted trend was found for precipitation, where richness in uncultivated sites declined as precipitation increased. Uncultivated soils contained approximately three‐fold more unique AM fungal species compared to cultivated soils. Our findings demonstrate that interactions between climate and land use strongly influence AM fungal biodiversity patterns in tropical mountain ecosystems. Incorporating both factors into conservation and sustainable agriculture strategies will be critical to preserving belowground biodiversity under global change.

## 1 Introduction

Land use change due to agricultural expansion is one of the largest contributors to biodiversity loss in terrestrial ecosystems globally (Ellis et al. 2010; Felipe‐Lucia et al. 2020; Newbold et al. 2015; Sala et al. 2000). This is particularly alarming in tropical mountain ecosystems, which cover only about 10% of Earth's surface but contain approximately 50% of biodiversity hotspots (Myers et al. 2000). The Andes in particular disproportionally contribute to the Earth's total biodiversity due to a high density of rare species found in the mountain range (Rahbek et al. 2019). Still, approximately 25–36 million hectares of uncultivated vegetation in the tropics were converted to agricultural land, often monoculture crops, between 2011 and 2015 (Pendrill et al. 2022). Studies of land use change and its effect on tropical biodiversity have historically focused on aboveground life (Guo et al. 2018; Peters et al. 2019). Consequently, the impacts on belowground life, where approximately 59% of all species reside, are poorly understood (Anthony et al. 2023; Guerra et al. 2020). Soil microbial diversity is particularly challenging to assess and often requires the use of molecular tools such as metabarcoding of environmental DNA to identify species. As a result, the effects of land use change on soil microbial diversity remain largely unknown in tropical mountain ecosystems.

Arbuscular mycorrhizal (AM) fungi are one of the most abundant groups of soil microbes, forming symbiosis with the roots of ~70% of terrestrial plant species, which in turn dominate Earth's biomass (Bar‐On et al. 2018; Brundrett and Tedersoo 2018; Smith and Read 2008). AM fungi can facilitate a significant proportion of plant nutrient uptake—up to 80% of a plant's phosphorus needs—in exchange for host carbon (Andrino et al. 2021; Barrett et al. 2011; Etesami et al. 2021; Leigh et al. 2009; Marschner and Dell 1994; Thirkell et al. 2016). The diversity of AM fungi in soils, both richness (number of species) and community composition, has been linked to ecosystem function as well, including plant productivity and nutrient cycling (Hawkins et al. 2023; Manoharan et al. 2017; Van Der Heijden et al. 1998, 2006; Wagg et al. 2011). Currently, we lack a complete understanding of how AM fungi are distributed in tropical ecosystems due in part to primer biases (Lekberg et al. 2018; Tedersoo et al. 2014) and poor representation of AM fungi in databases (Stockinger et al. 2010). Because of these biases and the relatively few soil samples collected in the tropics, it remains challenging to describe their AM fungal distributions accurately and their contributions to ecosystem functioning (Guerra et al. 2020; Stewart, Corrales, et al. 2025; Větrovský et al. 2023).

The impact of land use change on AM fungi may vary depending on local climate conditions (Peters et al. 2019). In the tropical Ecuadorian Andes, mean annual temperature (temperature) can decrease by as much as 0.5°C for every 100 m of elevational gain, resulting in significant climate variability over relatively short distances (Gorelick et al. 2017; Karger et al. 2017). This temperature gradient plays a critical role in shaping AM fungi diversity, with species richness often increasing and community composition shifting with temperature (Haug et al. 2019; Kivlin et al. 2011; Mikryukov et al. 2023; Van Nuland et al. 2025). Another important factor is mean annual precipitation (precipitation), as water availability also influences AM fungal diversity patterns (Mikryukov et al. 2023; van Galen et al. 2025; Van Nuland et al. 2025; Xi et al. 2022). These climate gradients further complicate comparisons of fungal diversity between sites as differences in diversity arise from differences in climatic conditions or through other processes unrelated to climate. In addition to climate, beta diversity can be influenced by nestedness, where communities at species‐poor sites contain subsets of species found at richer sites, and replacement, where species at one site are substituted by different species at another (Baselga 2010; Baselga and Baselga 2007).

Without understanding the impact of land use interactions with climate gradients, we risk overlooking areas to prioritise for soil conservation and restoration (Field et al. 2020; Guerra et al. 2020, 2022; Van Nuland et al. 2025). The conversion of uncultivated vegetation to monoculture cropland typically leads to reduced levels of AM fungal diversity (Banerjee et al. 2024; Edlinger et al. 2022; Guzman et al. 2021). For example, in Ecuador uncultivated grasslands harboured the highest AM fungal diversity, whereas just 200 m away cultivated soils showed varying effects (Avila‐Salem et al. 2020). Within cultivated lands, the addition of fertilisers often reduces AM fungal richness and alters community composition, depending on land management strategy (Babalola et al. 2022; Hijri et al. 2006). Together, changes in plant host diversity (e.g., multiple uncultivated hosts to monoculture crops) and agricultural inputs (e.g., fertilisers an pesticides) likely explain differences in AM fungal diversity patterns in cultivated soils compared to uncultivated soils (Guzman et al. 2021; Martín‐Robles et al. 2018; Ramana et al. 2023). Because climate gradients are superimposed over these land management practices, they further complicate our understanding of the drivers of AM fungal variation in soils, even in adjacent plots of land.

Comparing paired sites that vary in land use across climate gradients has previously helped clarify how climate and land use interact in other systems (Peters et al. 2019; Verbruggen et al. 2012). Because sites that differ in land use are located at similar positions (e.g., paired sampling sites) along the climate gradient, they allow direct comparison of land use effects within each climate zone. When these paired sampling protocols are repeated along the gradient, models can then estimate how climate and land use interact. For tropical Mt. Kilimanjaro, the impacts of land use change on species richness (of animals and plants) in farms and non‐agricultural land resulted in greater biodiversity loss in dry and hot regions of the mountain (Peters et al. 2019). In the tropics, the Andes mountains provide an ideal setting to test climate and land use interactions because of high AM fungal diversity, steep climate gradients over short distances and ongoing land use change (Curatola Fernández et al. 2015; Guarderas et al. 2022; Stewart, Corrales, et al. 2025; Van Nuland et al. 2025).

We tested the idea that land use and climate interact to shape the biogeography of tropical AM fungi. Specifically, we characterised AM fungal communities using metabarcoding in pairs of cultivated agricultural fields and adjoining uncultivated native vegetation. Our sampling transect covered two major climate gradients (temperature: 7.7°C–16.5°C and precipitation: 1000–3500 mm). With the resulting community diversity data, we asked (1) how does land use change from uncultivated to cultivated soils affect AM fungal community richness and composition? Previous research led us to hypothesize that richness would be reduced under cultivation and host distinct community compositions (Banerjee et al. 2024; Peng et al. 2024). Assuming that this is the case, we then aimed to further test (2) whether the magnitude and/or direction of observed land use change effects are related to the local climate of the sampling sites. We hypothesized that models which allow for land use effects to vary with climate would explain more variation than models with either climate or land use alone (Guo et al. 2018; Peters et al. 2019). Lastly, to gain further insight into the relative importance of different community assembly mechanisms underlying these patterns, we ask (3) what is the relative contribution of species richness, replacement and nestedness on changes in community composition between farms and surrounding uncultivated vegetation.

## 2 Materials and Methods

### 2.1 Soil Sampling

**FIGURE 1:**
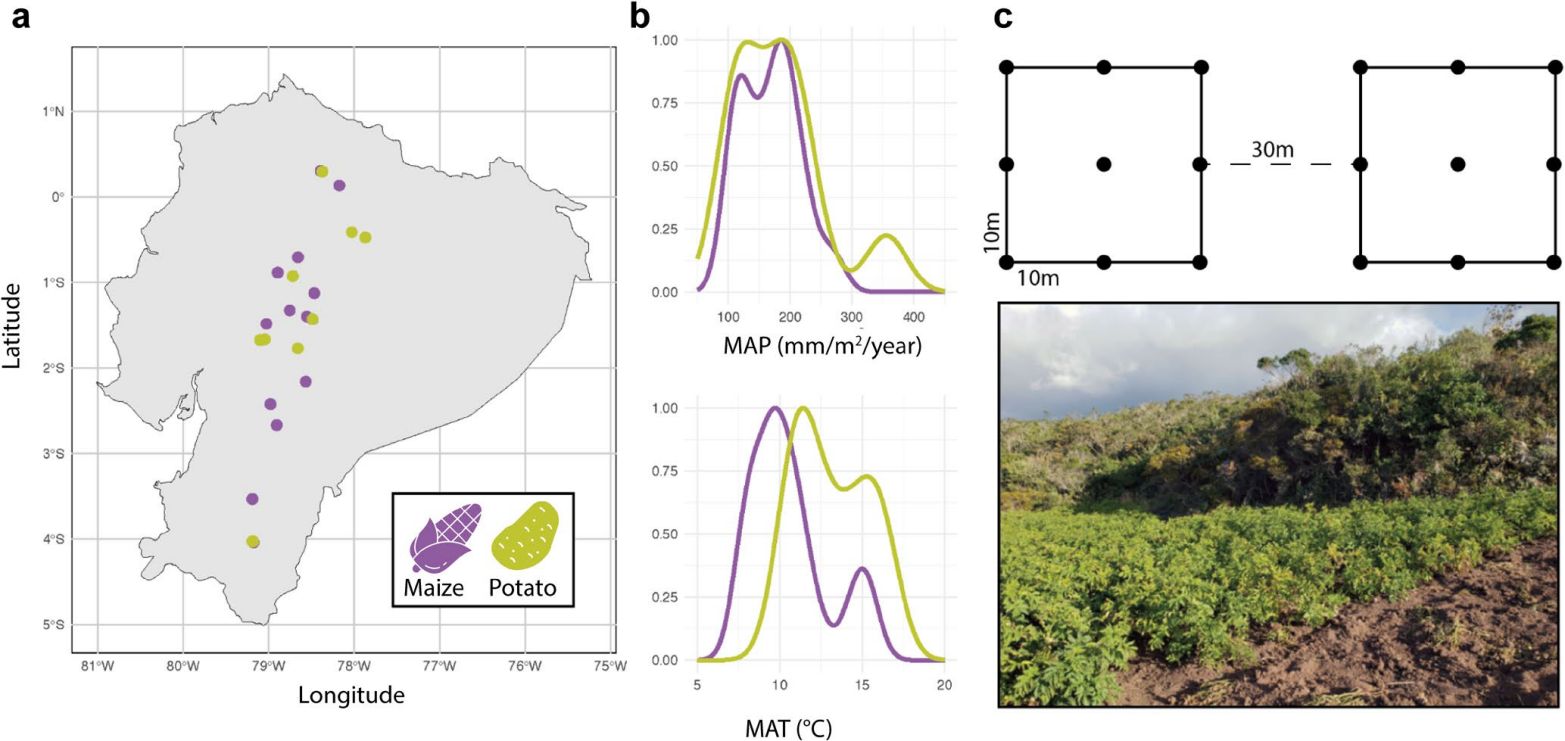
(a) Map of sampling locations in the Andean Mountain region of Ecuador. Samples were collected in either cultivated soils with either maize or potato crops, and in surrounding non‐agricultural uncultivated vegetation. Samples were collected along a climate gradient covering 1600 m in elevation across a ~500 km transect. (b) Frequency distributions of mean annual precipitation (MAP, mm/m^2^) and mean annual temperature (MAT, °C) at the sampling locations, colour coded by crop type. (c) Samples were collected in a 10m^2^ grid with nine subsamples collected at and between each vertex, and a center point. Subsamples were then homogenised into one bulk sample. The image shows an example of a potato farm in the foreground and uncultivated vegetation in the background.

We collected samples at 28 sites across the Andes Mountains region of Ecuador along a ~500 km transect from the northern to southern borders of the country in September 2023 (Figure 1). This transect included three ecoregions in the Ecuadorian Andes, namely the Northwest Andean montane forests, Eastern Cordillera Real montane forests and Northern Andean paramo (Dinerstein et al. 2017). After receiving permission from the landowners and stewards, we collected paired samples of farm (cultivated soil) and native vegetation (uncultivated soil), approximately 30–50 m away. Each composite sample was taken from a 10 × 10 m square plot, with nine subsamples taken from each vertex, the midpoint of each side and the central point, following a modification of the SoilBON protocol (Potapov et al. 2022). We then homogenised these nine samples in sterile bags by hand for 2 min. Next, we stored 100 g of soil in a 50 mL sterile plastic tube with silica gel for desiccation. At the end of each sampling day, we took the soil out of the tubes and air‐dried each sample overnight and froze it the following morning until downstream analysis (see DNA extraction and sequencing).

### 2.2 Soil Chemistry Analysis

We lyophilised a subset of each sample (~100 g) using a 2.5 L FreeZone dryer (Labconco) at −50°C and 0.01 mBar of pressure for 3 days. After drying, we ground and sieved the samples with a #35 mesh. We measured total nitrogen concentrations using the Kjeldahl method (BÜCHI Labortechnik AG), using urea as a control which showed an average recovery of 94%. For phosphorus, we mineralized the samples using a mix of nitric acid (67%) and hydrochloric acid (37%) (trace metal grade) and measured it using inductively coupled plasma‐optical emission spectroscopy (iCAP 7400 ICP‐OES spectrometer, Thermo Scientific). We prepared a calibration curve using Inorganic Ventures standard GCP 1 of 998 μg P/mL and established the wavelength following the EPA 6010 B method. The certified reference material San Joaquin soil (NIST 2709) was used for quality control, with an average recovery of 90%. We analysed total organic carbon using the combustion catalytic oxidation method in a TOC‐L analyser coupled with a solid sample combustion unit (SSM‐5000A, Shimadzu).

Dextrose served as the control, showing an average recovery rate of 95%. We next determined soil pH using a 1:2.5 soil to deionised water suspension, using a calibrated benchtop pH meter equipped with a glass electrode (Mettler Toledo). Calibration was performed using pH 4, 7 and 10 buffer standards. A summary of soil chemistry values is included in Table S1.

### 2.3 Remotely Sensed and Geospatial Data

We accessed data on temperature and precipitation gradients, as these are dominant drivers of both plant and AM fungal diversity patterns (Kokkoris et al. 2020; Sabatini et al. 2022; Tedersoo et al. 2014; Van Nuland et al. 2025). Specifically, we obtained mean annual temperature and mean annual precipitation rasters from the CHELSA dataset, ~1 km^2^ resolution at the equator (Karger et al. 2017). We selected CHELSA because it is widely used in climate–biodiversity studies, has been applied previously in the Andes, and is particularly suited for analyses in mountainous regions (Newell et al. 2022; Qin et al. 2025; Větrovský et al. 2019).

### 2.4 DNA Extraction and Sequencing

We extracted DNA from the thawed and sieved soil samples using the Qiagen DNeasy PowerSoil Pro Kit (Helden, Germany) with the protocol carried out according to the manufacturer's instructions including a negative extraction control. We amplified DNA using the AM fungi specific WANDA forward primer (5′—CAG CCG CGG TAA TTC CAG CT—3′) and reverse AML2 primer (5′—GAA CCC AAA CAC TTT GGT TTCC—3′), as described in (Kajihara et al. 2022). Amplicon sequencing was conducted by The Scripps Research Institute (California, USA) on the Illumina NextSeq 2000 platform at a sequencing depth of 150 K reads at the Genomics Core Facility. Upon receiving the DNA sequences, we used the Lotus2 (Özkurt et al. 2022) pipeline to carry out quality filtering and chimera removal. Together these steps led to 8,197,865 total reads after filtering.

We then used DADA2 (Callahan et al. 2016) to identify amplicon sequence variants (ASVs). Next, we followed the Lotus2 standard pipeline for filtering low frequency ASVs: ASVs present in only one sample must have ≥ 10 reads, ASVs present in two samples must have ≥ 5 reads each, and ASVs present in three samples must have ≥ 3 reads each. We further tested for contaminants using the *decontam* package in R with our negative control sample (Davis et al. 2017). Following this, we assigned taxonomy to ASVs using the BLAST search algorithm and the MaarjAM database (accessed May 2024) (Öpik et al. 2010) and SILVA (v138). We updated the MaarjAM database to correspond to Schussler's AM fungi taxonomy (http://www.amf‐phylogeny.com/). We estimated sample completeness using rarefaction curves which saturated for both land use types and crop hosts (Figure S1).

ASV richness was calculated using the Chao1 index for each sample using the *iNext* package (Hsieh et al. 2016). The Chao1 index is an abundance‐based estimator of species richness that adjusts for sampling depth (e.g., number of reads per sample) to approximate the true underlying diversity of a community and is commonly used in microbiome studies.

### 2.5 Modelling the Relationship Between Species Richness With Land Use, Climate and Soil Chemistry

We used a multimodel approach to identify how species richness relates to land use conversion along climate gradients, while controlling for soil chemistry differences (Appendix S1). A multimodel framework allows for comparisons of alternative hypotheses, helping to assess the relative importance of different climatic and environmental drivers of AM fungal richness. To handle such complex model structures, we applied a Bayesian modelling approach. Our approach included a set of models that progressively incorporated more complexity to explain variation in species richness (Table S2 for all model specifications). Starting with an intercept only model, we then added in covariates for soil chemistry, crop type, land use and climate; we built additive models that assume each factor (e.g., temperature, precipitation and land use) contributes independently to richness. The intercept only model assumes no predictor effects, serving as the null baseline for model comparison. We then developed interaction models that allowed climate variables (temperature and precipitation) to interact with land use. For the soil chemistry data, we tested models with each variable individually, but these showed poor fit (e.g., divergent transitions suggesting overparameterisation). To address this, we conducted a principal component analysis and included the first axis (PC1), which explained 51% of the variance in soil chemistry, including nitrogen, phosphorus, pH and soil organic carbon.

For all models, we included each site as a random intercept, to allow baseline levels of species richness to vary between sites. Additionally, each model was tested both with and without a random slope for land use. The random slope for land use was included to account for site‐specific practices (such as fertilisation or management differences by farmers) that may introduce unobserved variability across sites. We fit the models using the *brms* package with default priors on all parameters, running 3000 iterations across 4 chains, with a burn‐in of 500 iterations (Bürkner 2018). We compared the models using WAIC and *R* ^2^ values (Figure S3). Significance of model factors was assessed by calculating evidence ratios using the ‘hypothesis’ function in *brms* and generating probability values (*p*). Following standard practice, effects were considered significant if the 95% credible interval did not overlap zero. All other hypothesis testing produces *p*‐values which are reported with their associated test statistics (see below, e.g., PERMANOVA).

We also tested whether climate minima and maxima explained variation in AM fungal richness. Specifically, we extracted from the CHELSA dataset the mean daily maximum air temperature of the warmest month and the precipitation of the wettest month but found no significant relationships based on hypothesis testing. We obtained similarly insignificant results when using mean daily minimum air temperature of the coldest month and precipitation of the driest month. Across all climate by land use terms, the estimated coefficients showed wide uncertainty and each credible interval overlapped zero. The effects of mean daily maximum temperature of the warmest month (−5.87 ± 7.77), precipitation of the wettest month (11.16 ± 13.2), mean daily minimum temperature of the coldest month (1.78 ± 7.12) and precipitation of the driest month (1.1 ± 6.73) were therefore indistinguishable from being zero. We conclude that the climate by land use interactions were not significant in the minima/maxima models. All further analyses were conducted using mean annual values.

### 2.6 Modelling Changes in Community Composition of AM Fungi Along a Climate Gradient

We asked how climate, land use and soil chemistry influence AM fungi community composition. First, we calculated Bray–Curtis dissimilarity on our ASV matrix and performed a PERMANOVA (*n* = 1000) with the explanatory variables soil chemistry (PC1), mean annual temperature (°C), mean annual precipitation (mm/m^2^), land use and the location of each sample site (Site ID). Permutational tests with “betadispr” (*n* = 1000) were conducted to validate the assumption of homogeneity of variance for the PERMANOVA test.

Next, we visualised the AM fungal community composition using a distance‐based redundancy analysis. In our model, we included soil chemistry (PC1), mean annual temperature, land use and crop. Mean annual precipitation was excluded as it showed an insignificant relationship to community composition based on our PERMANOVA. Using the ‘envfit’ function in *vegan*, we fitted vectors onto the ordination where the length corresponds to variance explained and directionality the relationship with the ordination axes (Oksanen 2015).

Next, we asked what processes are associated with the differences in beta diversity between cultivated soils and soils adjacent to uncultivated vegetation, specifically the relative roles of species replacement and nestedness at each site. For this, we calculated the pairwise dissimilarity between each farm and uncultivated sample per site using the Sorenson index. We then decomposed the index into nestedness and replacement components using the ‘beta.pair’ function in *betapart* (Baselga and Orme 2012). This resulted in two numbers per site which contain the proportion of the dissimilarity between each pair of farmed and uncultivated sites that is attributed to either species replacement or nestedness. These numbers are additive and the sum of these two numbers is the total dissimilarity between the sites, and one minus this number is the residual dissimilarity from the index (Baselga 2010). These data were modelled using a Dirichlet regression with temperature and precipitation as independent variables, and the results were compared to an intercept‐only null model as well as models that included temperature or precipitation alone (Table S6). Soil chemistry was then included in our model as the log effect ratio of the N:P, C:N, pH in farm sites compared to uncultivated sites (e.g., log(N:P_farm_/N:P_uncultivated_)). All models were checked for convergence (all rhat = 1, mean bulk and tail effective sample sizes > 2000), and compared by WAIC values. We again used the *hypothesis* function to test the significance of model factors.

### 2.7 Identifying Exclusive ASVs to Crops and Land Uses

We identified exclusive ASVs for each land use category by filtering the ASV table to retain only taxa unique to cultivated or uncultivated soils. We then calculated the number of unique ASVs in each category and the relative differences between categories. This procedure was then repeated to identify exclusive ASVs between potato and maize farms.

## 3 Results

### 3.1 Biodiversity Loss Under Agricultural Land Use Is Climate Dependent

We collected a total of 56 samples across 28 farms cultivating maize or potato crops, together with paired surrounding soils (Figure 1). These samples covered a wide climatic gradient (mean annual temperature: 7.7°C–16.5°C, mean annual precipitation: 1000–3500 mm).

**FIGURE 2:**
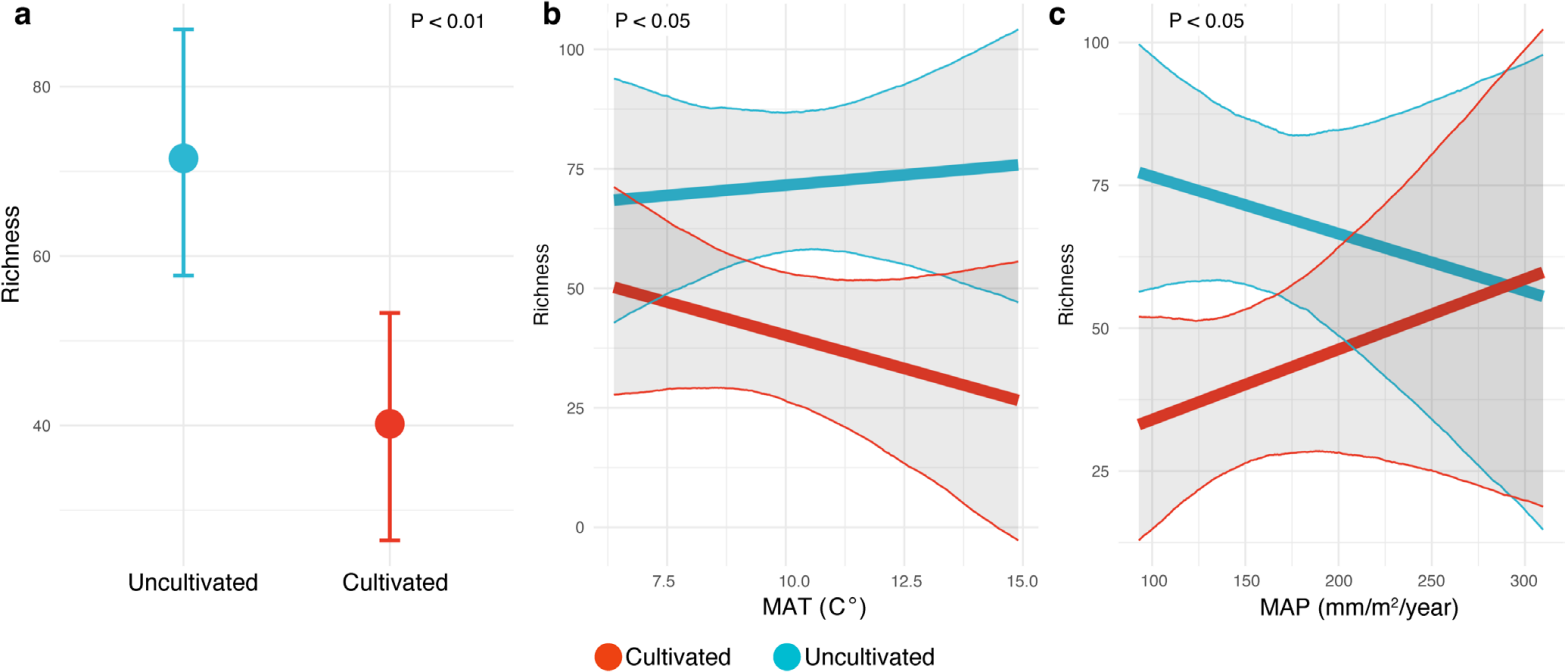
Impacts of land use and climate on arbuscular mycorrhizal (AM) fungi richness shown as the marginal effects of our best fitted model (Table S2, model #13). Richness was estimated using the Chao1 index applied to amplicon sequence variants (ASVs), which serve as proxies for species. Marginal effects are shown as they show the relationship averaged over the random effects of the model. Significance of our model coefficients were assessed by testing the hypothesis that model coefficients are equal to zero (a) Conversion of uncultivated vegetation to cultivated farmland reduced AM fungi richness (*p* < 0.05). Cultivated farms hosted on average 40 (± standard deviation 9) ASVs compared to soils with uncultivated vegetation which hosted an average 72 (± standard deviation 7) ASVs. (b) The interaction between land use and richness along the temperature (MAT) gradient was significant (*p* < 0.05). Along the temperature gradient, richness decreased in cultivated soils ~−9 ± 7 ASVs per unit standard deviation (2.6°C) and increased in adjacent cultivated fields ~2 ± 7. (c) Along a precipitation (MAP) gradient richness increased in farms and decreased in croplands (*p* < 0.05).

**FIGURE 3:**
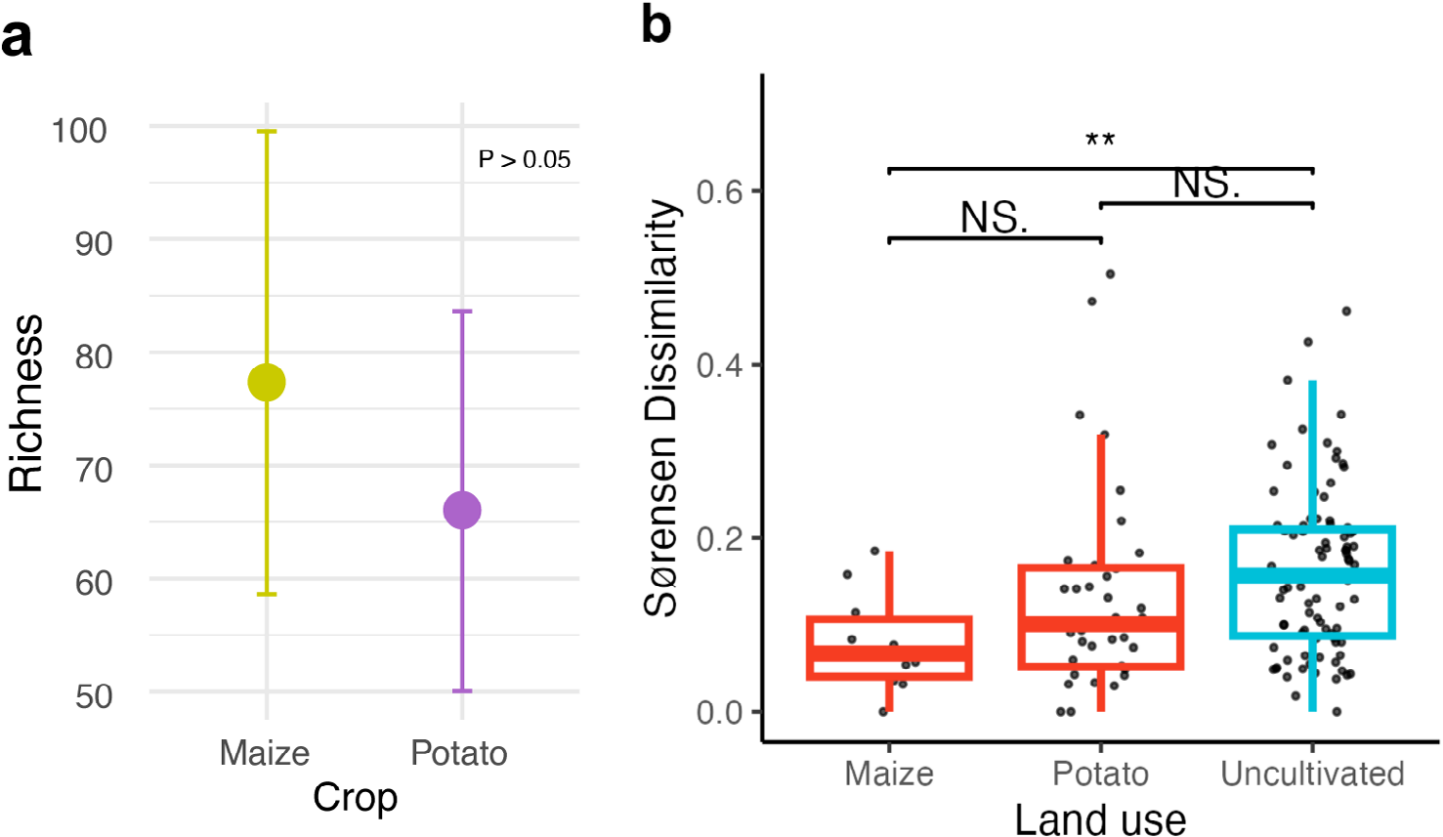
Differences in arbuscular mycorrhizal (AM) fungal communities according to crop type and landuse. (a) The marginal effects of the model predicting richness as a function of host crop, while controlling for differences in climate, land use, and soil chemistry. For this, the best fitted model was re‐fitted with crop as a fixed effect. There was not a significant difference in AM fungal richness between maize and potato farms (*p* > 0.05) (b) Pairwise similarities among plots with the same land‐use varied significantly between maize and uncultivated sites (*p* < 0.001). Maize and potato farms did not have significantly different dissimilarity values. Uncultivated sites were the most dissimilar to each other, and most variable.

First, we quantified AM fungal diversity using amplicon sequence variants (ASVs), which are unique DNA sequences resolved at single‐nucleotide differences and serve as proxies for microbial species. We then used these data to calculate species richness per site using the Chao1 index, a method that corrects for differences in abundances per sample and is commonly used in microbiome studies (Hsieh et al. 2016; Van Nuland et al. 2025). We found that soils supporting uncultivated vegetation exhibited higher richness of AM fungi compared to those in cultivated soils. An average of 72 (±7) ASVs were observed in soils with uncultivated vegetation, whereas soils from farms averaged 40 (±9) ASVs (Figure 2a). On average, this is an 80% increase in AM fungal richness in uncultivated soils relative to cultivated soils (*p* < 0.01). Species richness did not display significant variation between maize and potato fields (Figure 3a).

We then used a series of statistical models to evaluate the relative influence of climate and land use on AM fungal richness. These models assessed whether climate and land use effects on AM fungal richness were additive or if an interaction between these factors had a more significant impact on richness patterns (Table S2). Among the 13 models tested, the best‐fitting model specified interactions between land use and both temperature and precipitation, along with additive effects of soil chemistry (Table S2, model #13). The model incorporated a random intercept for each site and a random slope for land use, yielding a ∆WAIC of approximately −11 compared to the intercept‐only model and an *R* ^2^ of 0.56 (Figure S4). The inclusion of the random effects in this model also accounts for unmeasured site‐specific variation, for example, agricultural practices (e.g., tillage and fertilisation). The marginal effects of this model showed that cultivated and uncultivated sites displayed contrasting relationships between climate variables and AM fungal richness (Figure 2B,C). Along the temperature gradient, richness decreased in cultivated soils (~ −3 ± 2 ASVs per unit increase 1°C, Table S3) and increased in adjacent cultivated fields (~1 ± 3 ASVs per unit increase 1°C, Figure 2B, *p* < 0.05 for difference between slopes, Table S3). Similarly, AM fungal richness along the precipitation gradient differed between uncultivated and cultivated soils. AM fungal richness increased in cultivated soils (~6 ± 4 ASVs per 10 mm precipitation) and decreased in cultivated soils (~−3 ± 4 along the precipitation gradient) (Figure 2C, *p* < 0.05 for difference between slopes, Table S3).

### 3.2 Community Composition of AM Fungi Is Altered by Land Use Change

We found that community composition of AM fungi varied significantly between land‐use types and along climatic gradients. Uncultivated sites exhibited the highest community dissimilarity, followed by potato sites, and then maize sites (Figure 3b).

**FIGURE 4:**
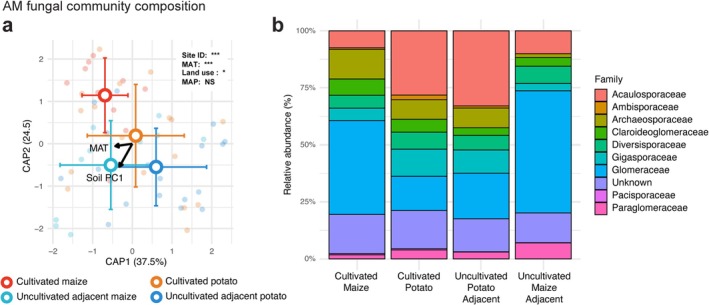
The impacts of environmental variables and land use on arbuscular mycorrhizal (AM) fungi community composition. (a) Distance‐based redundancy analysis (CAP) of the AM fungi communities found in all sites, based on Bray–Curtis dissimilarity. We found that temperature, land use, soil chemistry and site location (sampling location along transect) all showed a significant effect on community composition (total variance explained = 62.9%, all *p* < 0.05). When tested with a sequential permutational test (*n* = 1000). The plot is coloured by land use (blues = soils with uncultivated vegetation, reds = cultivated soils), and arrow length and direction represent the relative importance and directionality of the variable on community composition. (b) Composition barplots showing the mean relative abundance of the AM fungal families identified in our samples.

To further assess these patterns, we applied distance based redundancy analysis to relate environmental variation patterns in community composition. This analysis revealed a significant combined effect of climate and land use on AM fungal community composition compared to a null model (Figure 4a, *p* = 0.016) Further testing using PERMANOVA (Table S4) identified significant effects of sampling location along the transect which explained the most variance in AM fungal community composition (*r* ^2^ = 0.50, *p* = 0.002), followed by land use (*r* ^2^ = 0.06, *p* = 0.046), soil chemistry (*r* ^2^ = 0.03, *p* = 0.008) and temperature (*r* ^2^ = 0.03, *p* = 0.016). Precipitation did not have a significant effect on community composition (*p* = 0.18). To test the homogeneity of multivariate dispersions (Table S5), the variance around group centroids was assessed using beta dispersion analysis. The analysis indicated no significant differences in variance among the groups for soil chemistry (*p* = 0.14) and land use (*p* = 0.52), but was significant for temperature (*p* = 0.007).

Community composition varied significantly between the uncultivated sites surrounding farms and the farms themselves (Figure 4b). Cultivated maize soils were dominated by Glomeraceae (44.2%), whereas adjacent uncultivated vegetation showed an even higher proportion (53.6%). In contrast, potato farms were dominated by Acaulosporaceae (28.2%), and this family was more abundant in the uncultivated sites adjacent to potato farms (32.9%). Glomeraceae were less common in potato fields (15.0%) but increased to 20.0% in adjacent uncultivated soils.

Next, we assessed the presence of unique ASVs, defined as taxa found only within a specific land use and vegetation type (e.g., maize farms), across all samples. Soils with uncultivated vegetation hosted approximately three times more exclusive ASVs compared to cultivated soils (Figure S2a). A total of 316 ASVs were identified as unique to soils with uncultivated vegetation, whereas only 110 ASVs were exclusive to cultivated farm soils. The AM fungi taxa unique to uncultivated soils were primarily associated with the genera *Glomus* (31.6%), *Acaulospora* (19.0%) and *Paraglomus* (9.1%). Comparing between cultivated sites, potato farms hosted significantly distinct communities of unique AM fungi compared to maize farms (Figure S2b). Approximately twice as many exclusive ASVs were observed in potato farms (210 ASVs) compared to maize farms (95 ASVs). The AM fungi taxa unique to uncultivated soils were predominantly from the genera *Glomus* (31.6%), *Acaulospora* (19.0%) and *Paraglomus* (9.1%).

### 3.3 Species Replacement Underlies Compositional Differences Between Land Use Types

Next, we quantified the proportion of community dissimilarity associated with nestedness (where less diverse communities are subsets of more diverse ones) and replacement (also referred to as turnover, where ASVs in one land use are replaced by ASVs in another, such as between farms and uncultivated sites). To assess these components, we calculated nestedness and replacement indices at each site by decomposing the Sorenson index, comparing each farm to its paired, adjacent uncultivated vegetation site. The Sorenson index measures similarity between two communities based on species composition. We then fitted two Dirichlet regressions, which model compositional data, to test the additive and interactive effects of temperature and precipitation on beta diversity between sites, with soil chemistry differences included as a control variable. These models estimated three parameters: dissimilarity due to nestedness, dissimilarity due to species turnover and residual dissimilarity. The best fit model included the additive effects of temperature and precipitation (Table S6).

**FIGURE 5:**
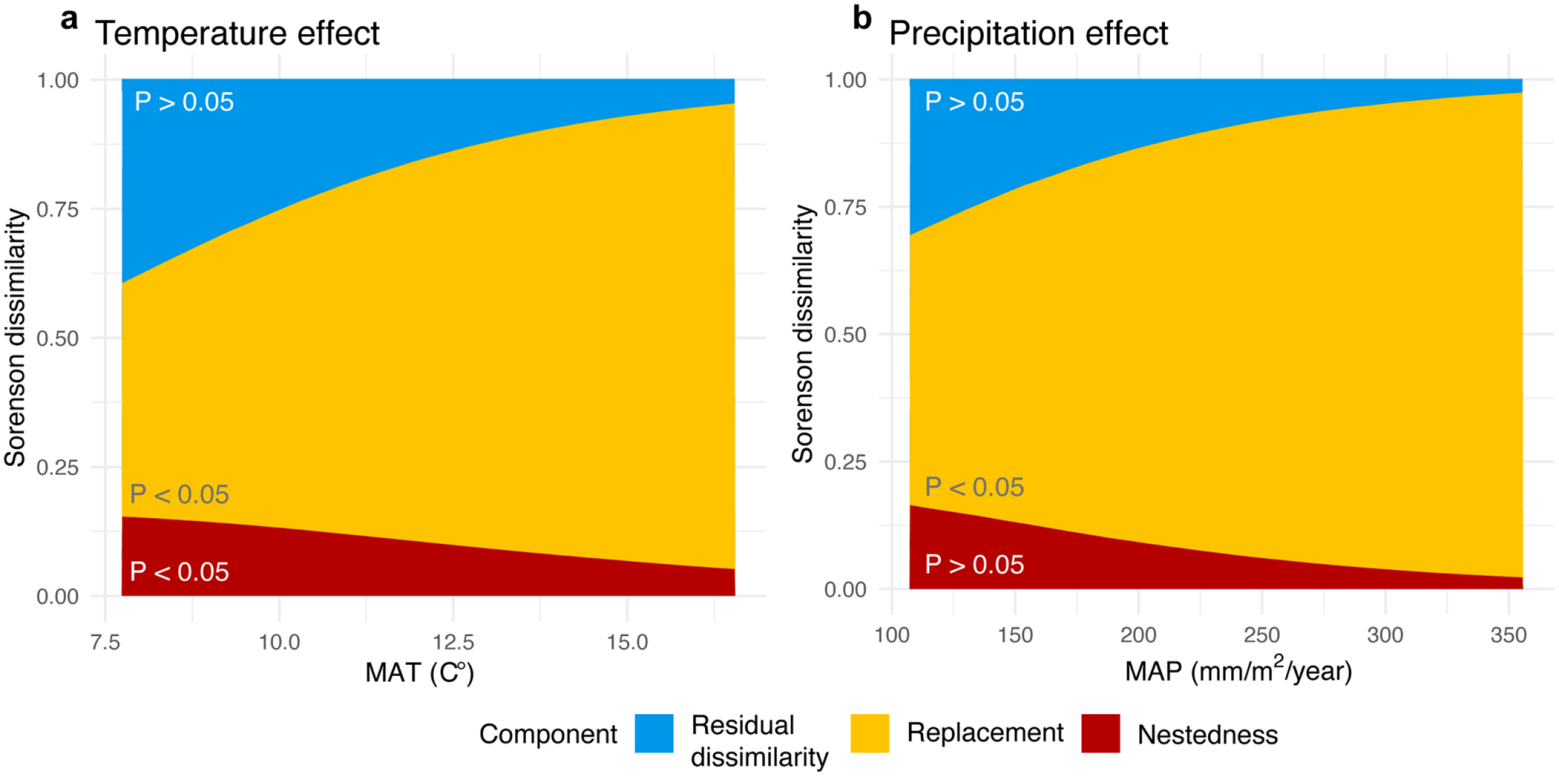
Marginal effects from the best fitted Dirichlet regression model correlating pairwise beta diversity in arbuscular mycorrhizal (AM) fungi communities in soils between individual farms and their surrounding uncultivated vegetated areas with climate variables. These plots show the relative proportion of dissimilarity, which sums to one, attributed to replacement (center, yellow) and nestedness (bottom, red), and the residual dissimilarity (top, blue) (a) The marginal effects for temperature (MAT, *°C*) show that along the temperature gradient that communities of AM fungi became less similar, largely due to species replacement. Species replacement increased along the temperature gradient. (b) Along the precipitation (MAP, mm/m^2^) gradient species replacement was the dominant process contributing to the Sorenson dissimilarity index. The role of species replacement increased with increasing precipitation, with the proportional decrease of similarity and nestedness. Significance of model coefficients was tested using the *hypothesis* function to estimate the probability the coefficient is not equal to zero (e.g., no effect).

Species replacement was the dominant process explaining differences in AM fungal communities between farms and uncultivated sites, as indicated by the decomposition of the Sorensen index (Figure 5a). Averaged across the temperature and precipitation gradients, species replacement accounted for 64.2% of the Sorenson index. This means that the communities in farms and adjacent uncultivated areas are largely composed of different species, rather than being subsets of each other. In contrast, nestedness, where less diverse communities in farms are subsets of more diverse communities in uncultivated areas, on average accounted for 10.2% of the Sorenson index. In other words, the shifts between farms and uncultivated areas are not just due to reduced diversity but also involve substantial species turnover.

The relative roles of species replacement and nestedness varied across both the temperature and climate gradients. As temperature increased, the contribution of species replacement to community change increased. In contrast, the role of nestedness decreased with increasing temperature (Figure 5a, *p*_replacement_ < 0.05, *p*_nestedness_ < 0.05, Table S6). Similar to the temperature gradient, along the precipitation gradient, the role of species replacement increased, while nestedness did not significantly vary (Figure 5b, *p*_replacement_ < 0.05, *p*_nestedness_ > 0.05, Table S6).

## 4 Discussion

The interaction between climate and land use strongly shaped the biogeography of soil AM fungi diversity in a tropical mountain ecosystem. AM fungal richness varied between cultivated and uncultivated sites but did not differ significantly between maize and potato farms. On average, cultivated sites hosted 40 (± SD 9) ASVs compared to soils with uncultivated vegetation with 72 (± SD 7) ASVs. We found that uncultivated soils also supported approximately three‐fold more exclusive ASVs compared to farms, with 316 unique ASVs in uncultivated soils versus 110 unique ASVs in cultivated farm soils. Maize and potato farms differed in community composition, with potato farms showing greater similarity to uncultivated vegetation than maize farms.

Climate and land use interactions explained 56% of the variation in AM fungal richness across a large temperature and precipitation gradient, while accounting for differences in soil chemistry and unmeasured site specific variation (e.g., management and fertilisation). Along both the temperature and precipitation climate gradients, cultivated and uncultivated land use types displayed opposite relationships with AM fungal richness. In soils with uncultivated vegetation, richness increased with temperature and decreased with precipitation. In contrast, AM fungal richness in cultivated soils was negatively correlated with the temperature gradient and positively correlated with the precipitation gradient. Variation in community composition was largely driven by spatial species turnover, rather than nestedness. Spatial turnover occurs when species in one site are replaced by different species in another site, whereas nestedness occurs when communities in species poor sites are subsets of communities in richer sites. The proportional contributions of these two processes were correlated with temperature and precipitation gradients. Higher turnover was found at hotter and wetter sites, alongside a proportional decline in nestedness. These results show that the effects of land use on AM fungal community composition and richness are strongly shaped by climate in these tropical mountain ecosystems.

### 4.1 Host Identity Influences AM Fungal Community Composition

AM fungal diversity is often strongly influenced by the diversity of host plants, as demonstrated in this and other studies (Klironomos et al. 2000; Van Der Heijden et al. 1998). These findings align with our first hypothesis that uncultivated vegetation would host higher AM fungal diversity than cultivated fields. Experimental work has shown that soils with greater plant species diversity tend to support higher AM fungal richness (Van Der Heijden et al. 1998). Further, the choice of crop species in a farmer's field impacts AM fungal community composition, with fields that are less diverse in host plants often hosting less diverse communities of AM fungi (Guzman et al. 2021).

A limitation is that we were unable to identify the plant species present in the uncultivated sites. This is particularly difficult in the Andes, which are recognised as plant biodiversity hotspots (Sabatini et al. 2022). The complexity of these plant communities makes it challenging to document all species present at a site. This limited our ability to determine whether wild relatives of potato or other members of the Solanaceae were present. If such relatives were growing nearby, uncultivated soils might host AM fungal communities that are more similar to those in cropland, while their absence could lead to larger shifts in community composition upon land use change (Davison et al. 2020; Dong et al. 2021). In this way, information on which plant species occur at what locations on climate gradients and how they relate to crop lineages could influence interpretations of similarities between uncultivated and cultivated soils. Identifying the local plant community would also help link plant traits to shifts in AM fungal diversity (Bergmann et al. 2020; Deveautour et al. 2018; Ramana et al. 2023). Future research should therefore examine the local plant composition of uncultivated sites to better quantify drivers of variation in AM fungal diversity.

### 4.2 Climate and Land Use Interactions Shape Biodiversity Patterns

We identified that precipitation exerted a strong influence on AM fungal richness, with cultivated and uncultivated soils showing opposing trends. At global scales, AM fungal richness often follows a hump‐shaped relationship with precipitation (Mikryukov et al. 2023). At local scales, precipitation has been associated with both increases and decreases in AM fungal richness, depending on site conditions and vegetation structure (Gao et al. 2016; Sousa et al. 2022; Zhang et al. 2016). In our study, we found AM fungal richness increased with precipitation in cultivated soils but decreased in uncultivated soils. One possible explanation is that higher precipitation may cool exposed cultivated soils, allowing fungi that are otherwise thermally stressed to persist. In contrast, uncultivated vegetation buffers soil temperature and moisture, promoting stable microclimates that may already sit near the upper bound of moisture tolerance for many AM fungal taxa. In such contexts, additional precipitation could push soils toward overly wet conditions, reducing AM fungal colonisation and contributing to declines in richness (Deepika and Kothamasi 2015; Miller and Sharitz 2000).

Land use also shaped how temperature related to AM fungal richness patterns. Evidence from elevational studies shows that microclimatic variation related to vegetation structure, which is also correlated to temperature patterns, strongly influences both plant‐associated bacteria and soil fungi (Ma et al. 2022; Schoenborn et al. 2023). Structural equation modelling studies have also shown that temperature is a predictor of AM fungal community composition (Chai et al. 2018). In our study, cultivated soils likely experienced higher temperature variability due to reduced shade and greater exposure following vegetation removal, which can increase soil temperatures (Defries et al. 2002). These conditions may push AM fungal taxa outside their thermal niches and contribute to declines in richness along the temperature gradient in cultivated fields (Davison et al. 2021). Uncultivated vegetation, by contrast, often buffers solar radiation and cools soils by several degrees (Chen et al. 1999). This buffering could allow a given thermal niche to cover a greater geographic range and may explain the positive richness and temperature relationship we observed in these sites.

Together, these findings support the idea that microclimatic shifts associated with land use are a major driver of divergent biodiversity patterns along climatic gradients. Cultivated soils often lose the shading, canopy structure and continuous root cover that stabilise both temperature and moisture microclimates. Periods of planting, harvest and fallow intensify this instability, contributing to rapid temperature and moisture fluctuations (Cach‐Pérez et al. 2021). In contrast, uncultivated vegetation cools soils by more than 5°C and enhances moisture retention by up to 45% (Chen et al. 1999; Pérez‐Hernández et al. 2021). These contrasting microclimates may provide an explanation for why cultivated and uncultivated soils show opposing richness‐climate relationships but was not possible due to the resolution of climate dataset. Testing these mechanisms more directly will benefit from new remote sensing tools, including LIDAR‐based vegetation mapping, which can resolve fine‐scale differences in canopy structure and microclimate heterogeneity (Zellweger et al. 2019).

### 4.3 Differences in Community Composition Between Land Use Types Are Driven by Species Replacement

Species replacement, as opposed to nestedness, was the dominant driver of differences in AM fungal community composition between cultivated farms and adjacent uncultivated vegetation. Rates of species replacement were highest at the hottest and wettest sites, with a proportional decrease in the role of nestedness. This contrasts with other paired‐design studies of AM fungi in contrasting land use types, where species poor agricultural fields were found to be nested subsets of nearby species rich fields, in a temperate region with low climatic variability (Verbruggen et al. 2012). The high turnover of AM fungi diversity in uncultivated soils compared to farm soils mirrors that of aboveground organisms in the tropics, attributed to narrower thermal tolerances and limited dispersal capabilities (McCain 2009; Polato et al. 2018; Shah et al. 2017). Compared to regions with low climatic variation, this results in high biodiversity with rapid turnover in community composition (Allen and Gillooly 2006; Mittelbach et al. 2007). In other studies excluding fungi, the interaction between land‐use change and elevation has been shown to influence species ranges. These shifts, driven by variations in mean annual temperature, result in diverging community compositions between land use types (Guo et al. 2018).

If AM fungi in the tropics are highly endemic or localised, even small shifts in environmental conditions may lead to species turnover, compared to nestedness. If microclimates are driving differences in community composition, this may lead to farm soils excluding species from the uncultivated vegetation, rather than simply retaining a subset of them. This environmental filtering may promote replacement of species in uncultivated soils with narrow climatic niches, such as rare AM fungal taxa. Rare AM fungi are often more geographically isolated, while generalists are more widespread (Kajihara et al. 2022). Further, agricultural soils often host fewer rare AM fungal taxa in exchange for a higher abundance of generalists (Banerjee et al. 2024; Trevizan Chiomento et al. 2019). An additional possibility is that some AM fungal taxa may have been introduced with crop planting material, which could contribute to species replacement in cultivated soils.

We also found ~three‐fold more ASVs only detected in uncultivated sites, compared to agricultural soils. If agricultural soils are dominated by generalist AM fungi, while uncultivated soils host a higher proportion of rare AM fungal taxa, soil microclimates may play a key role in driving differences in community composition, as has been seen for other groups of organisms (Ma et al. 2022; Schoenborn et al. 2023). These factors together could explain why differences in community composition between farmed and uncultivated soils are largely driven by species replacement rather than nestedness.

### 4.4 Broader Implications

We found that land use and climate interactions reshaped AM fungal biodiversity patterns in uncultivated soils, leading to lower biodiversity in cultivated soils. Given the impact of climate‐land use interactions, land management strategies could potentially leverage these dynamics to identify areas where restoration efforts may contribute the most to restoring degraded soils (Andean Mountain Initiative 2024; United Nations FAO 2002). This is particularly important because recent work has revealed systematic biases against the protection of soil biodiversity, with AM fungi among the least protected groups of soil life (Bothe et al. 2010; Qin et al. 2025; Van Nuland et al. 2025).

A more complete ecological understanding of how soil life is distributed also requires integrating interactions between AM fungi and the broader soil microbiome, if microbes that may have been introduced with crops. Other fungal guilds (e.g., decomposers) and bacteria can influence AM fungal communities through both cooperation and competition for soil resources and plant hosts, which may vary across different geographic regions (Frey 2019; Hestrin et al. 2019; Kakouridis et al. 2024; Klein et al. 2021; Lahrach et al. 2024; Stewart, Kiers, et al. 2025). Such interactions are likely to also differ between cultivated and uncultivated environments, which may also explain differences in diversity patterns (Banerjee et al. 2024; Peng et al. 2024). Incorporating these considerations into future work will provide a more complete picture of how climate and land use shape belowground biodiversity in tropical mountain ecosystems.

## Author Contributions

J.D.S. conducted field work, analyses and writing the first draft. D.X.R., A.L.‐R., N.B. and S.L. conducted field work. B.F.M. conducted bioinformatic analyses. N.C.‐S. and M.R.‐U. assisted with soil chemistry analyses. All authors contributed to writing.

## Funding

NWO Gravity Grant MICROP [024.004.014] (J.D.S., E.T.K., J.T.W., D.X.R. and J.M.R.), SPUN is funded with grants from the Jeremy and Hannelore Grantham Environmental Trust, the Paul Allen Family Foundation, the Bezos Earth Fund, the Schmidt Family Foundation and the Hefner Foundation (J.D.S., B.F.M. and E.T.K.).

## Conflicts of Interest

The authors declare no conflicts of interest.

## Supporting information


**Appendix S1:** mec70253‐sup‐0001‐AppendixS1.docx.

## Data Availability

The data that support the findings of this study are openly available in Github at https://github.com/thecrobe/EcuadorAMF.
