# Supplementary material for: Land Use Change Reshapes Climate‐Driven Diversity Patterns of Tropical Arbuscular Mycorrhizal Fungi
Source: Mol Ecol. 2026 Jan 28;35(2):e70253. doi: 10.1111/mec.70253 (PMC12853079; doi:10.1111/mec.70253)
Supplement: Supplementary file 1 — Appendix S1: mec70253‐sup‐0001‐AppendixS1.docx. [file MEC-35-e70253-s001.docx]

**Supplemental information:**

**Supplemental File 1:** CSV file containing information on site coordinates and sample metadata (soil chemistry, climate variables, land use).

**Table S1:** Summary of soil chemistry per land use and crop plant. Here the results are shown averaged and with the standard deviation per group.

| **Land use** | **Crop** | **Nitrogen % (mean)** | **Nitrogen % (sd)** | **Total organic carbon % (mean)** | **Total organic carbon % (sd)** | **Phosphorus mg per liter (mean)** | **Phosphorus mg per liter (sd)** |
| --- | --- | --- | --- | --- | --- | --- | --- |
| Farm | Maize | 0.08 | 0.04 | 3.67 | 2.33 | 1074.18 | 831.23 |
| Farm | Potato | 0.12 | 0.07 | 5.42 | 3.34 | 1107.37 | 955.08 |
| Uncultivated | Maize | 0.12 | 0.10 | 6.28 | 5.06 | 557.77 | 369.43 |
| Uncultivated | Potato | 0.13 | 0.10 | 7.32 | 5.63 | 561.47 | 235.00 |

**Table S2:** A table presenting the models fitted to correlate richness with land use, crop, soil chemistry, and climate. Models were fitted and compared using Watanabe-Akaike information criterion (WAIC) and R^2^ values to select the best model, which is the model (#13) with the formula: Climate * land use + soil chemistry, with a random intercept for each site and random slope for landuse. MAP is mean annual precipitation and MAT is mean annual temperature. Site_ID is the location of each pairedsampling site.

| **Model** | **Formula** | **Random effects** |
| --- | --- | --- |
| Intercept (#1) | Chao1~1+(1\|Site_ID) | Intercept: Site |
| Crop (#2) | Chao1~Crop+(1\|Site_ID) | Intercept: Site |
| Land use (#3) | Chao1~Land_use+(1\|Site_ID) | Intercept: Site |
| Soil chemistry (#4) | Chao1~CN+NP+pH+(1\|Site_ID) | Intercept: Site |
| Climate (#5) | Chao1~MAP+MAT+(1\|Site_ID) | Intercept: Site |
| Climate + land use (#6) | Chao1~MAP+MAT+Land_use+(1\|Site_ID) | Intercept: Site |
| Climate + land use + soil chemistry (#6) | Chao1~MAP+MAT+Land_use+CN+NP+pH+(1\|Site_ID) | Intercept: Site |
| Climate * land use + soil chemistry (#7) | Chao1~ +MAP*Land_use+CN+NP+pH+(1\|Site_ID) | Intercept: Site, Slope: Land use |
| Intercept (#8) | Chao1~1+(1+Land_use\|Site_ID) | Intercept: Site, Slope: Land use |
| Crop (#9) | Chao1~Crop+(1+Land_use\|Site_ID) | Intercept: Site, Slope: Land use |
| Land use (#10) | Chao1~Land_use+(1+Land_use\|Site_ID) | Intercept: Site, Slope: Land use |
| Soil chemistry (#11) | Chao1~CN+NP+pH+(1+Land_use\|Site_ID) | Intercept: Site, Slope: Land use |
| Climate (#11) | Chao1~MAP+MAT+(1+Land_use\|Site_ID) | Intercept: Site, Slope: Land use |
| Climate + land use (#11) | Chao1~MAP+MAT+Land_use+(1+Land_use\|Site_ID) | Intercept: Site, Slope: Land use |
| Climate + land use + soil chemistry (#12) | Chao1~MAP+MAT+Land_use+CN+NP+pH+(1+Land_use\|Site_ID) | Intercept: Site, Slope: Land use |
| Climate * land use + soil chemistry (#13) | Chao1~MAT*Land_use+MAP*Land_use+CN+NP+pH+(1+Land_use\|Site_ID) | Intercept: Site, Slope: Land use |

**Table S3:** Results of nonlinear hypothesis testing for the Bayesian richness models. The posterior probability calculated using the evidence ratio represents the proportion of posterior samples consistent with a hypothesis. Here the land use and climate interaction hypothesis was testing for differences being coefficient slopes, and for land use testing if the coefficient overlaps zero. A posterior probability greater than 0.95 is considered significant, meaning that a hypothesis is rejected less in than 5% of all posterior samples. A posterior probability greater than 0.95 indicates strong hypothesis support, and are reported in the main text as P < 0.05.

| **Effect** | **Evidence Ratio** | **Posterior Probability (“P”)** |
| --- | --- | --- |
| Temperature x Land use | 17.75 | 0.955 |
| Precipitation x Land use | 62.49 | 0.984 |
| Land use (no interaction) | 626.45 | 0.998 |

**Table S4:** Results of the permutational ANOVA (PERMANOVA), with all model terms and associated statistics shown. Mean annual precipitation was evaluated but was not significant (p=0.18) and was removed from the final model. 1000 permutations were used for the PERMANOVA.

| **Term** | **Df** | **Sum of Squares** | **R²** | **F-Statistic** | **p-value** |
| --- | --- | --- | --- | --- | --- |
| Soil variables PC1 | 1 | 0.6053 | 0.03465 | 2.3471 | 0.008099 |
| Mean annual temperature | 1 | 0.5458 | 0.03125 | 2.1166 | 0.015699 |
| Land use | 3 | 1.1013 | 0.06304 | 1.4236 | 0.046000 |
| Site_ID | 25 | 8.7696 | 0.50201 | 1.3603 | 0.002300 |
| Residual | 25 | 6.4468 | 0.36905 |  |  |
| Total | 55 | 17.4688 | 1.00000 |  |  |

**Table S5:** Results of the dispersion tests for homogeneity of variance, with all model terms and associated statistics shown. 1000 permutations were used for these tests.

| **Factor** | **Df** | **Sum Sq** | **Mean Sq** | **F-Statistic** | **p-value** |
| --- | --- | --- | --- | --- | --- |
| Land use | 3 | 0.02944 | 0.0098148 | 0.8391 | 0.524 |
| Residuals | 52 | 0.60824 | 0.0116968 |  |  |
| **MAT** | 23 | 0.61660 | 0.0268088 | 3.1267 | 0.007 |
| Residuals | 32 | 0.27437 | 0.0085741 |  |  |
| **Soil chemistry (PC1)** | 3 | 0.04869 | 0.016230 | 1.8352 | 0.141 |
| Residuals | 52 | 0.45989 | 0.008844 |  |  |

**Table S6:** Results of nonlinear hypothesis testing for the Bayesian richness models. The posterior probability calculated using the evidence ratio represents the proportion of posterior samples consistent with a hypothesis. Here the hypothesis was testing for if model coefficients overlapped with zero. A posterior probability greater than 0.95 is considered significant, meaning that a hypothesis is rejected less in than 5% of all posterior samples. A posterior probability greater than 0.95 indicates strong hypothesis support, and are reported in the main text as P < 0.05.

| **Effect** | **Evidence Ratio** | **Posterior Probability (“P”)** |
| --- | --- | --- |
| Replacement Temperature | 624.0 | 0.998 |
| Nestedness Temperature | 25.5 | 0.962 |
| Nestedness Precipitation | 2.42 | 0.708 |
| Replacement Precipitation | 832.3 | 0.998 |
| Residual Temperature | 1.30 | 0.565 |
| Residual Precipitation | 2.11 | 0.677 |


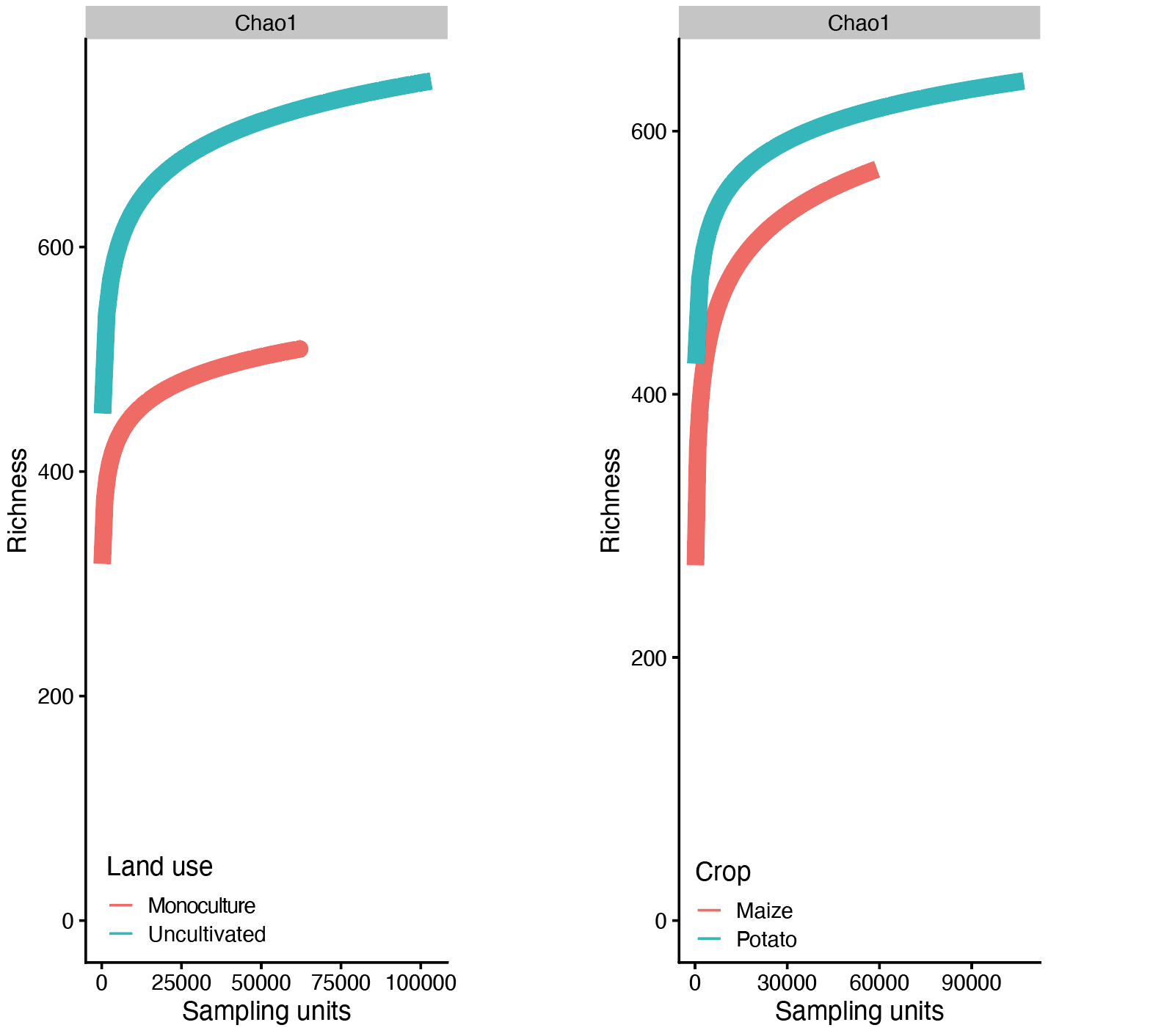


**Figure S1:** Rarefaction curves estimating the sampling completeness of our sequencing efforts, grouped by land use and crop. Here all curves have reached asymptotes, indicating we have deeply sampled the communities. This means all or most of the arbuscular mycorrhizal (AM) fungi in our samples are likely represented in our analyses.


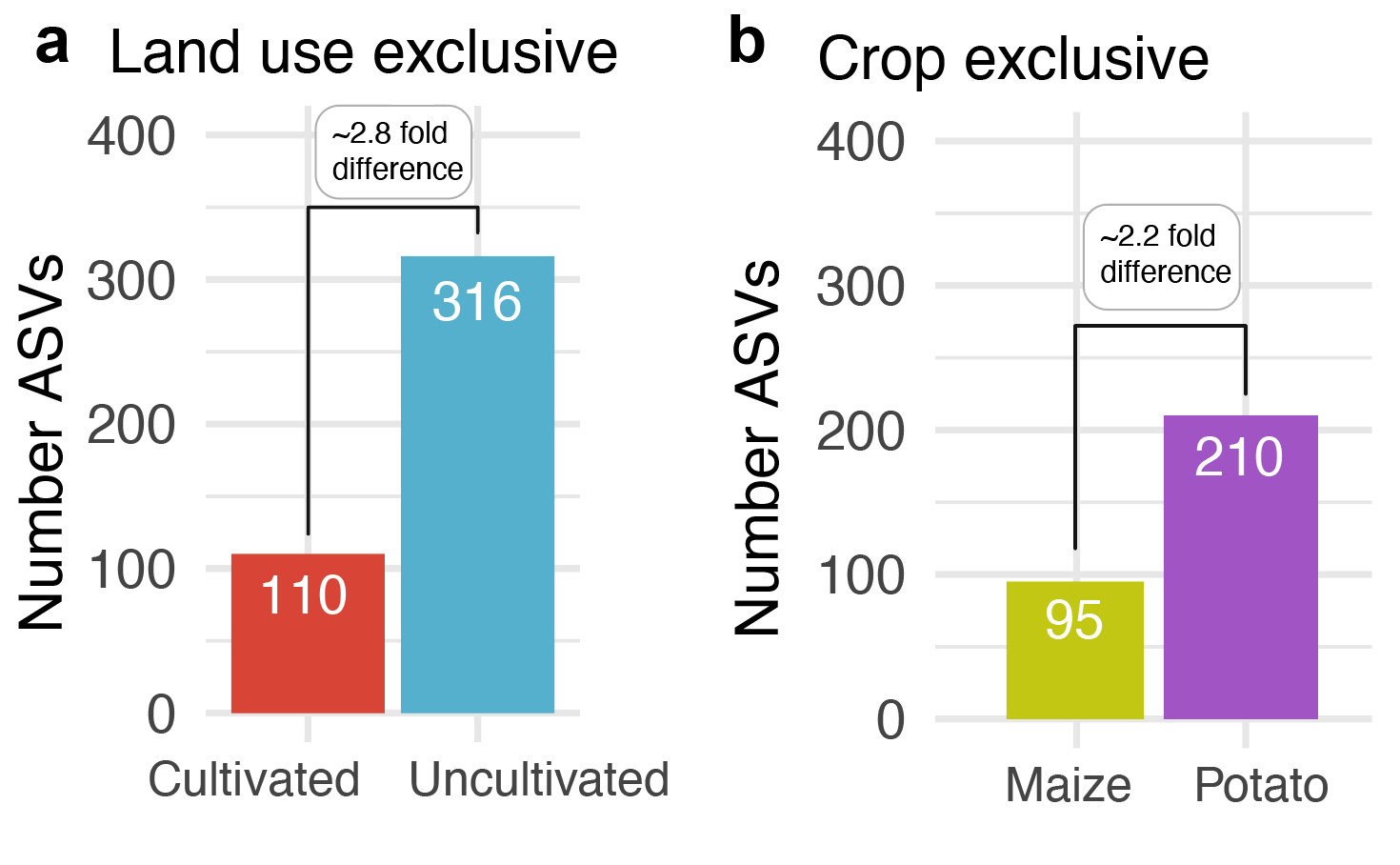


**Fig. S2.** Amplicon sequence variants (ASVs) exclusive to crops and land use types. Exclusive ASVs are ASVs found to a single category. **a)** The number of exclusive ASVs varies between maize and potato farms. Maize farms hosted 95 exclusive ASVs not found in potato farms, while potato farms hosted 210 ASVs absent from maize farms. There is an ~ 2.2 fold difference in the number of exclusive ASVs between maize and potato farms. **b)** Uncultivated vegetation hosted 316 exclusive ASVs while cultivated farms (both maize and potato) 110 exclusive ASVs. This represents an approximate ~2.8 fold difference in the number of exclusive ASVs between land use types.


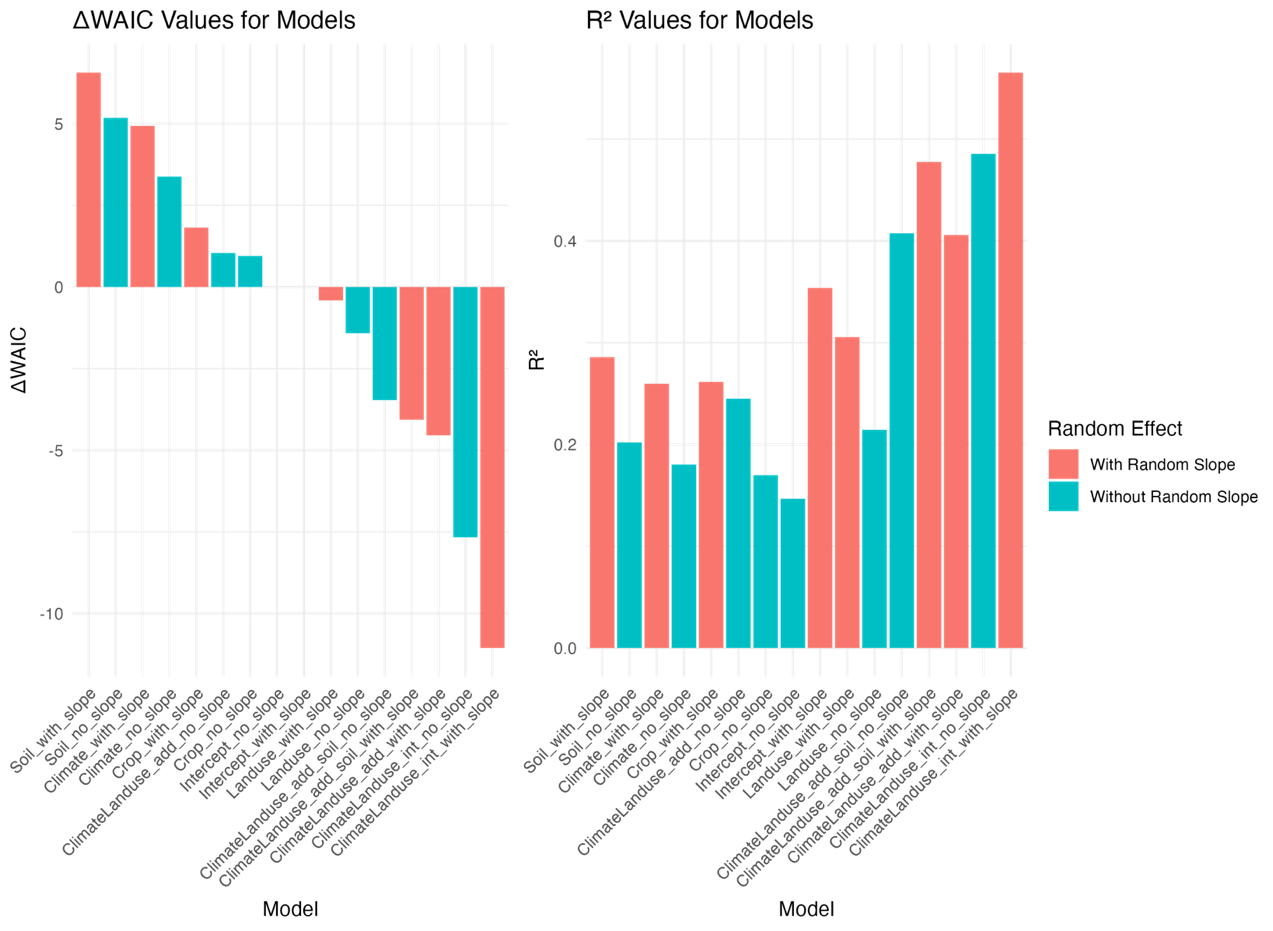


**Fig. S3:** Model selection metrics. This figure presents two bar plots assessing model performance in predicting Chao1 richness estimates. The models are categorized by whether they include land use as a random slope ("With Random Slope") or not ("Without Random Slope"), with blue indicating models without the random slope and red for those with the random slope included. The left plot shows change in Watanabe-Akaike information criterion (WAIC) values, indicating the model fit relative to an intercept-only model, where lower values signify better fit. The right plot displays R² values, reflecting the proportion of variance explained by each model, with higher values indicating stronger explanatory power. Together, these plots reveal the impact of including land use as a random slope on both model fit and explanatory capacity.

**
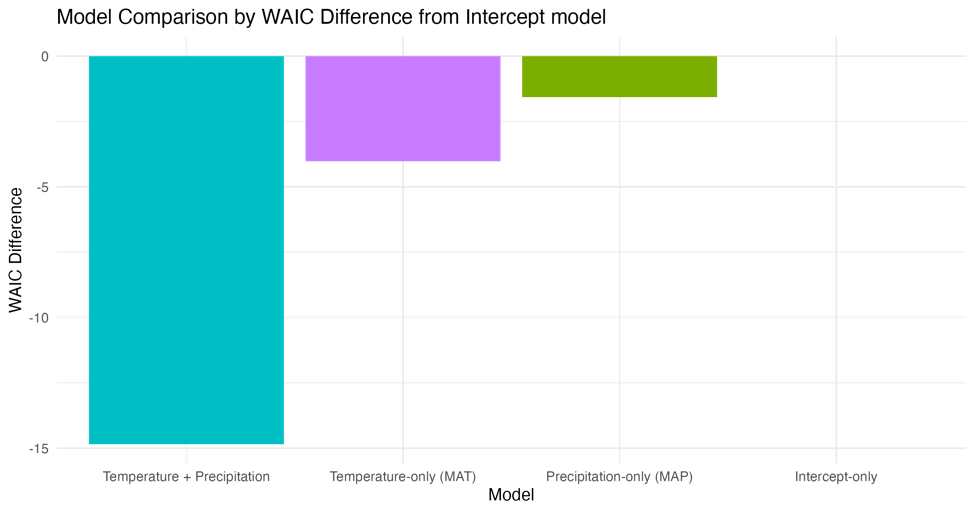
**

**Fig. S4:** Dirichlet model comparisons using Watanabe-Akaike information criterion (WAIC) values. Here the plot shows the change in WAIC value compared to the intercept only model, where lower values signify better fit.
